# Role of HPV 16 variants among cervical carcinoma samples from Northeastern Brazil

**DOI:** 10.1186/s12905-020-01035-0

**Published:** 2020-08-01

**Authors:** Rodrigo Lopes da Silva, Zulmira da Silva Batista, Gerusinete Rodrigues Bastos, Ana Paula Almeida Cunha, Fábio Vidal Figueiredo, Lailson Oliveira de Castro, Liwerbeth dos Anjos Pereira, Marcos Antonio Custódio Neto da Silva, Flávia Castello Branco Vidal, Maria Claudene Barros, Elmary da Costa Fraga, Luciane Maria Oliveira Brito, Maria do Carmo Lacerda Barbosa, Miguel Ângelo Martins Moreira, Maria do Desterro Soares Brandão Nascimento

**Affiliations:** 1 Federal University of Maranhão (UFMA), São Luís, Maranhão Brazil; 2 Faculty of Medicine, Federal University of Maranhão (UFMA), São Luís, Maranhão Brazil; 3 Faculty of Medical Sciences (FCM), State University of Campinas (UNICAMP), Campinas, São Paulo Brazil; 4 Center of Advanced Studies of Caxias (CESC), State University of Maranhão (UEMA), Caxias, Maranhão Brazil; 5 Genetic Division, National Institute of Cancer (INCA), Rio de Janeiro, Brazil

**Keywords:** Cervical cancer, Human papillomavirus, HPV 16, Variants

## Abstract

**Background:**

Cervical cancer is the fourth most common type of cancer affecting women globally. In Brazil, it is the third most frequent type of cancer in women and HPV is present in approximately 90% of cases. Evidence suggests that variants of HPV 16 can interfere biologically and etiologically during the development of cervical cancer.

**Methods:**

Cervix tumor fragments were collected, their DNA was extracted, and nested PCR was used to detect HPV. Positive samples were sequenced to determine the viral genotype. To characterize the HPV 16 strains, positive samples PCR was used to amplify the LCR and E6 regions of the HPV 16 virus.

**Results:**

Data from 120 patients with cervical cancer were analyzed. Most women were between 41 and 54 years of age, had schooling until primary school, a family income between 1 and 2 times the minimum wage and were married/in a consensual union. There was no statistically significant association between HPV or socio-demographic variables and risk factors for cervical cancer (*P* <  0.05). HPV was present in 88 women (73%). The most prevalent types were HPV 16 (53.4%), HPV 18 (13.8%), HPV 35 (6.9%) and HPV 45 (5.7%). Of the 47 HPV 16 positive cases, variant A (49%) was present in 23 samples, followed by variant D in 20 cases (43%), and variants B and C in 2 cases each (4%). The most prevalent histological type of HPV 16 tumors was squamous cell carcinoma, followed by adenocarcinoma. There was a statistically significant association between HPV 16 variants and the tumors’ histological types (*P* <  0.001).

**Conclusions:**

Knowledge of HPV 16 variants will provide data on their influence on the pathological and oncogenic aspects of cervical lesions.

## Background

Cervical cancer is the fourth most common cancer type affecting women worldwide, with more than 265,000 estimated deaths annually and more than 80% of cases occurring in developing countries [1, 2]. According to INCA, cervical cancer is the third most common cancer type in the Brazilian female population. They estimate that there will be 16,370 new cervical cancer cases for the biennium from 2018 to 2019 [3].

It is estimated that in the state of Maranhão there will be around 1090 new cervical cancer cases for 2018, with240 of them the capital, São Luís, with a gross rate of incidence of 30.55 cases per 100,000 inhabitants [3].

Human Papillomavirus (HPV) is the main etiological factor for cervical cancer and is associated with the development of diseases ranging from benign warts to invasive cancer [4]. Although approximately 80% of women acquire HPV infection by the age of 50, less than 1% of persistent infections progress to invasive cervical cancer [5].

The reason why only some uterine cervical lesions associated with high-risk genotypes progress to invasive cancer remains unknown. Evidence suggests that variants of the same HPV type can interact biologically and etiologically during cancer development [6, 7].

The HPV16 is the most carcinogenic HPV type followed by HPV18 and most infections are asymptomatic [8]. A few studies have been conducted in Brazil on HPV 16 variants. This type of study has never been conducted in Maranhão. Interest in this topic has been growing in recent years, with variation in carcinoma prognosis at different stages of the disease depending on the viral variant found attracting special interest. Even in research carried out other places in the world, many of these questions remain unclear and many of the findings are contradictory, making it necessary to develop new and continuous approaches to this topic.

Despite of the relationship between different HPV type and the development of cancer being well established, evidence suggests that genetic variations between the same viral type may influence infection potential, viral persistence, the development of precursor lesions and the progression to invasive cancer [9–12]. Due to the high prevalence of HPV 16 in cases of cervical cancer, the association between HPV 16 and cancer has been studied at the level of intratype variants, and several lines of study have attributed a higher risk of invasive cervical cancer to some HPV 16 strains.

Studies on HPV variants have been developed with the objective of understanding their association with pathological and oncogenic aspects of cervical lesions. Factors influencing HPV infection in cervical cancer are not completely clear, but it is believed that HPV 16 variants playa fundamental role in cervical carcinogenesis and are currently recognized as an important marker for research on viral transmission, persistence and carcinogenicity [5]. Variation in these aspects may contribute to disparities in cervical cancer incidence.

Regarding these aspects, the aim of this study was to evaluate the prevalence of HPV 16 variants in cervical carcinoma samples from Northeastern, Brazil. This is the first evaluation of genetic diversity of HPV 16 in the State of Maranhão, Brazil.

## Methods

### Type of Study

This is descriptive, prospective, and transversal study.

#### Period and location of study

The study was conducted from January 2016 to December 2017, at the High Complexity Care Unit (UNACON) of the Cancer State Hospital of Maranhão and in the High Complexity Care Center in Oncology (CACON) of the Aldenora Bello Cancer Hospital. This work was approved by the Research Ethics Committee of the Federal University of Maranhão (CEP-UFMA), under Consolidated Opinion N° 1.289.419/2015.

#### Population and sample

The study population was composed of 120 women with a diagnosis of cervical cancer, who were treated at the previously mentioned hospitals.

##### Inclusion criteria

Women older than 18 years diagnosed with cervical cancer who agreed to participate in the study by signing an Informed Consent Form (ICF).

##### Exclusion criteria

Women who presented with surgical indications as an initial treatment or who presented with small lesions, in which biopsy could interfere with staging. Women in psychiatric treatment were also excluded.

#### Collection instrument and data evaluation

Initially, the patients were referred for outpatient care at the Oncology Gynecology Service of the Cancer State Hospital of Maranhão and the Aldenora Bello Cancer Hospital and were invited to participate in the study by signing an Informed Consent Form (ICF). A questionnaire was supplied to collect socio-demographic, reproduction, and smoking data.

Further, women were submitted to gynecological examination. A cervical biopsy was done and cervical tumor fragments were then placed in microtubes containing 1 mL of RNA Later solution (Life Technologies) at 4 °C and were transported in thermal boxes to the Multiuser Laboratory in the Biobank of Tumors and DNA of Maranhão of the Federal University of Maranhão, University Hospital of the Federal University of Maranhão (HUUFMA). After 24 h, the samples were removed from the RNA Later solution and stored in a freezer at − 80 °C until use.

### Experimental procedures

#### Detection of HPV DNA

The extraction of the genomic DNA from the samples was performed using the QIAamp DNA FFPE Tissue Purification Kit (QIAGEN®) according to manufacturer protocols.

The Nested PCR reactions were performed by using primers PGMY09 and PGMY11 for the first round, and primers GP + 5 and GP + 6 for the second round [12] (Table 1).

**Table 1:** Primer sequences used for PCR reaction to identify HPV DNA

***Primer***	Sequence 5′ - 3′	
**PGMY11**	PGMY11-A	GCA CAG GGA CAT AAC AAT GG
	PGMY11-B	GCG CAG GGC CAT AAT AAT GG
	PGMY11-C	GCA CAG GGA CAT AAT AAT GG
	PGMY11-D	GCC CAG GGC CAC AAC AAT GG
	PGMY11-E	GCT CAG GGT TTA AAC AAT GG
**PGMY09**	PGMY09-F	CGT CCC AAA GGA AAC TGA TC
	PGMY09-G	CGA CCT AAA GGA AAC TGA TC
	PGMY09-H	CGT CCA AAA GGA AAC TGA TC
	PGMY09-Ia	G CCA AGG GGA AAC TGA TC
	PGMY09-J	CGT CCC AAA GGA TAC TGA TC
	PGMY09-K	CGT CCA AGG GGA TAC TGA TC
	PGMY09-L	CGA CCT AAA GGG AAT TGA TC
	PGMY09-M	CGA CCT AGT GGA AAT TGA TC
	PGMY09-N	CGA CCA AGG GGA TAT TGA TC
	PGMY09-Pa	G CCC AAC GGA AAC TGA TC
	PGMY09-Q	CGA CCC AAG GGA AAC TGG TC
	PGMY09-R	CGT CCT AAA GGA AAC TGG TC
	HMB01b	GCG ACC CAA TGC AAA TTG GT
**GP + 5/6**	GP + 5	TTT GTT ACT GTG GTA GAT ACT AC
	GP + 6	GAA AAA TAA ACT GTA AAT CAT ATT C

#### Sanger sequencing method

HPV genotypes were determined by Sanger sequencing method using a1000 MegaBACE sequencer (GE Healthcare, UK) at the Molecular Biology Laboratory of the State University of Maranhão located in the Center of Higher Studies of Caxias (CESC-UEMA).

Sequencing was performed with an ET Dye Terminator Cycle Sequencing Kit (GE Healthcare, UK), according to the manufacturer’s protocol.

The Chromas program was used to obtain electropherograms of the HPV DNA sequences present in the samples. To identify the HPV type the nucleotide sequences were compared to the Genbank Nucleotide Sequence Database using the BLAST program (NCBI).

#### Identification of HPV 16 variants

After determination of the HPV 16 strains, the LCR regions and E6 virus gene of the positive samples were amplified with specific primers. The reaction mixture consisted of a final volume of 25 μL, with 1X PCR Buffer, 2.5 mM of MgCl_2_, 0.25 μM of each dNTP, 100 pmol/L of each primer, 50–100 ng of DNA, and 2.5 U of Platinum Taq Polymerase. The PCR reaction consisted of denaturation at 95 °C for 10 min, followed by 40 cycles of 95 °C for 1 min; annealing temperature for 1 min and 72 °C for 1 min, followed by a final extension step for 15 min.

The PCR product was subsequently purified and sequenced according to the above protocol. The consensus sequences were merged using the Geneious software (Biomatters Ltd.) and all the sequences generated were aligned with HPV 16 specific strains, using the reference sequences proposed by Burk et al. [13], using the MEGA Software (version 6.0, www.megasoftware.net). BLAST online (Available at: http://www.ncbi.nlm.nih.gov/blast/Blast.cgi) was then used to identify the HPV types.

### Phylogenic analysis

The phylogenetic tree of the HPV 16 strains was constructed from the 1300 bp sequences from the E6 and LCR regions using the “neighbor joining” method with p-distance (obtained with pairwise deletion) using the program Mega 4.1. The references proposed by Burk et al. (2013) [13] were included. The analysis of the HPV 16 variants was performed at the National Cancer Institute (INCA) under the supervision of Dr. Miguel Ângelo Martins Moreira.

We have provided GenBank accession numbers for nucleotide sequences: MT568878 to MT568924.

### International Federation of Obstetrics and Gynecology (FIGO) system for cervical cancer

The cervical carcinoma samples were classified according to FIGO staging system [14].

### Statistical analyses

Descriptive statistical analysis was performed using the Stata program (version 14.0). The x^2^ (Chi-square) test was used to verify the association between HPV and sociodemographic and clinical variables, and *P* values ≤0.05 were considered statistically significant. The values corresponding to don’t know/did not respond were excluded from the association analysis.

## Results

### Sociodemographic and clinical data

Among the women diagnosed with cervical cancer, HPV was present in 88 (73.3%). The majority of these women were in the age group 40 to 49 years of age (28.3%), and self-declared with mixed-race (70%), had schooling up to elementary school (42.5%), had a family income between 1 and 2 times the minimum wage (55%) and were married or in a consensual union (51.7%). There was no statistically significant association between the sociodemographic variables and the presence of HPV (*p* < 0.05) (Table 2).

**Table 2:** Association between sociodemographic factors and the presence of HPV

Sociodemographic Variables	HPV					
	Total	Negative		Positive		***p***-value
	N(%)	n	%	N	%	
**Age (Years)**						
≤ 29	8 (6.7)	4	50.0	4	50.0	0.421
30 to 39	20 (16.7)	5	25.0	15	75.0	
40 to 49	34 (28.3)	9	26.5	25	73.5	
50 to 59	19 (15.8)	7	36.8	12	63.2	
60 to 69	19 (15.8)	4	21.0	15	79.0	
≥ 70	20 (16.7)	3	15.00	17	85.00	
**Ethnicity**						
European-descent	20 (16.7)	3	15.00	17	85.00	0.377
African-descent	13 (10.8)	2	15.4	11	84.6	
East Asian- descent	3 (2.5)	1	33.3	2	66.7	
Mixed-race	84 (70)	26	31.0	58	69.0	
**Marital Status**						
Single	39 (32.5)	12	30.8	27	69.2	0.774
Married/Consensual Union	62 (51.7)	14	22.6	48	77.4	
Divorced/Separated	6 (5)	2	33.3	4	66.7	
Widow	13 (10.8)	4	30.8	9	69.2	
**Family Income**						
Less than the minimum wage	29 (24.2)	7	24.1	22	75.9	0.797
1 to 2 times the minimum wage	66 (55)	18	27.3	48	72.7	
Above 2 times the minimum wage	20 (16.7)	4	20.00	16	80.00	
Don’t Know/Didn’t Answer	5 (4.2)	3	60.00	2	40.00	
**Education**						
None	41 (34.2)	13	31.7	28	68.3	0.399
Adult Education	4 (3.3)	0	0.00	4	100.0	
Primary Education/Primary Level	51 (42.5)	11	21.6	40	78.4	
High School/Secondary Level	20 (16.7)	8	40.00	12	60.00	
Higher Incomplete	1 (0.8)	0	0.00	1	100.0	
Higher Complete	1 (0.8)	0	0.00	1	100.0	
Don’t Know/Didn’t Answer	2 (1.7)	0	0.00	2	100.0	

Of the risk factors associated with cervical cancer, for most women first sexual intercourse occurred between 10 and 19 years of age (72.5%), a first pregnancy between 16 and 21 (34.2%) and 1 to 3 children (30.8%) were most common. Having only one sexual partner during their lifetime (31.7%) was most common followed by more than 3 partners (30%). Most women reported never having used contraceptive methods (58.3%) (Table 3).

**Table 3:** Association between cervical cancer risk factors, reproductive history and presence of HPV

Risk Factors	HPV						
	Total		Negative		Positive		***p***-value
	n	%	N	%	N	%	
**Onset of sexual activity (years)**							
10 to 19	87 (72.5)		25	28.7	62	71.3	0.738
20 to 29	9 (7.5)		3	33.3	6	66.7	
Above 30	2 (1.7)		0	0.00	2	100.0	
Don’t Know/ Did not answer	22 (18.3)		4	18.2	18	81.8	
**Age at 1st pregnancy**							
11 to 15	11 (9.2)		1	9.1	10	90.9	0.268
16 to 21	41 (34.2)		14	34.1	27	65.9	
22 to 27	13 (10.8)		5	38.5	8	61.5	
Above 27	2 (1.7)		0	0.00	2	100.0	
Don’t Know/ Did not answer	53 (44.2)		12	22.6	41	74.0	
**Number of pregnancies**							
1 to 3	37 (30.8)		11	29.73	26	70.3	0.859
4 to 6	26 (21.7)		6	23.1	20	76.9	
7 to 9	32 (26.7)		8	25.0	24	75.0	
10 to12	13 (10.8)		4	30.8	9	69.2	
Above 12	8 (6.7)		1	12.5	7	87.5	
Don’t Know / Did not answer	4 (3.3)		2	50.0	2	50.0	
**Use of contraceptive**							
Yes. Actually Use	7 (5.83)		2	28.57	5	71.43	0.887
Yes. Already Used	40 (33.33)		12	30.00	28	70.00	
No	70 (58.33)		18	25.71	52	74.29	
Don’t Know /Did not answer	3 (2.50)		0	0.00	3	100	
**Number of sexual partners**							
1	38 (31.7)		11	28.9	27	71.1	0.406
2	27 (22.5)		4	14.8	23	85.2	
≥ 3	36 (30.0)		8	22.2	28	77.8	
Don’t know/Did not answer	19 (15.8)		9	47.4	10	52.6	

Most women (70%) reported having undergone examination before their diagnosis with cervical cancer and most (36.7%) said they received preventive exam annually. Most women reported no tobacco use (57.5%) (Table 4).

**Table 4:** Association between preventive examination, smoking and the presence of HPV

Risk Factors	HPV						
	Total		Negative		Positive		***p***-value
	n	%	n	%	n	%	
**Preventive examination before diagnosis**							
No	29	(24.2)	12	41.4	17	58.6	0.070
Yes	84	(70.0)	20	23.8	64	76.2	
Don’t Know/ Did not Answer	7	(5.8)	0	0.00	7	100.0	
**Frequency of Pap test**							
Every Year	44	(36.7)	12	27.3	32	72.7	0.153
Every 2 Years	9	(7.50)	1	11.1	8	88.9	
Every 3 Years	1	(0.8)	1	100.0	0	0.0	
4 years or more	1	(0.8)	1	100.0	0	0.0	
Without Regularity	32	(26.7)	8	25.0	24	75.0	
Don’t Know/ Did not answer	33	(27.5)	9	27.3	24	72.7	
**Smoking**							
No	69	(57.5)	22	31.9	47	68.1	0.208
Yes	43	(35.8)	9	20.9	34	79.1	
Don’tKnow/ Did not answer	8	(6.7)	1	12.5	7	87.5	

### Identification of HPV type

**Fig. 1:**
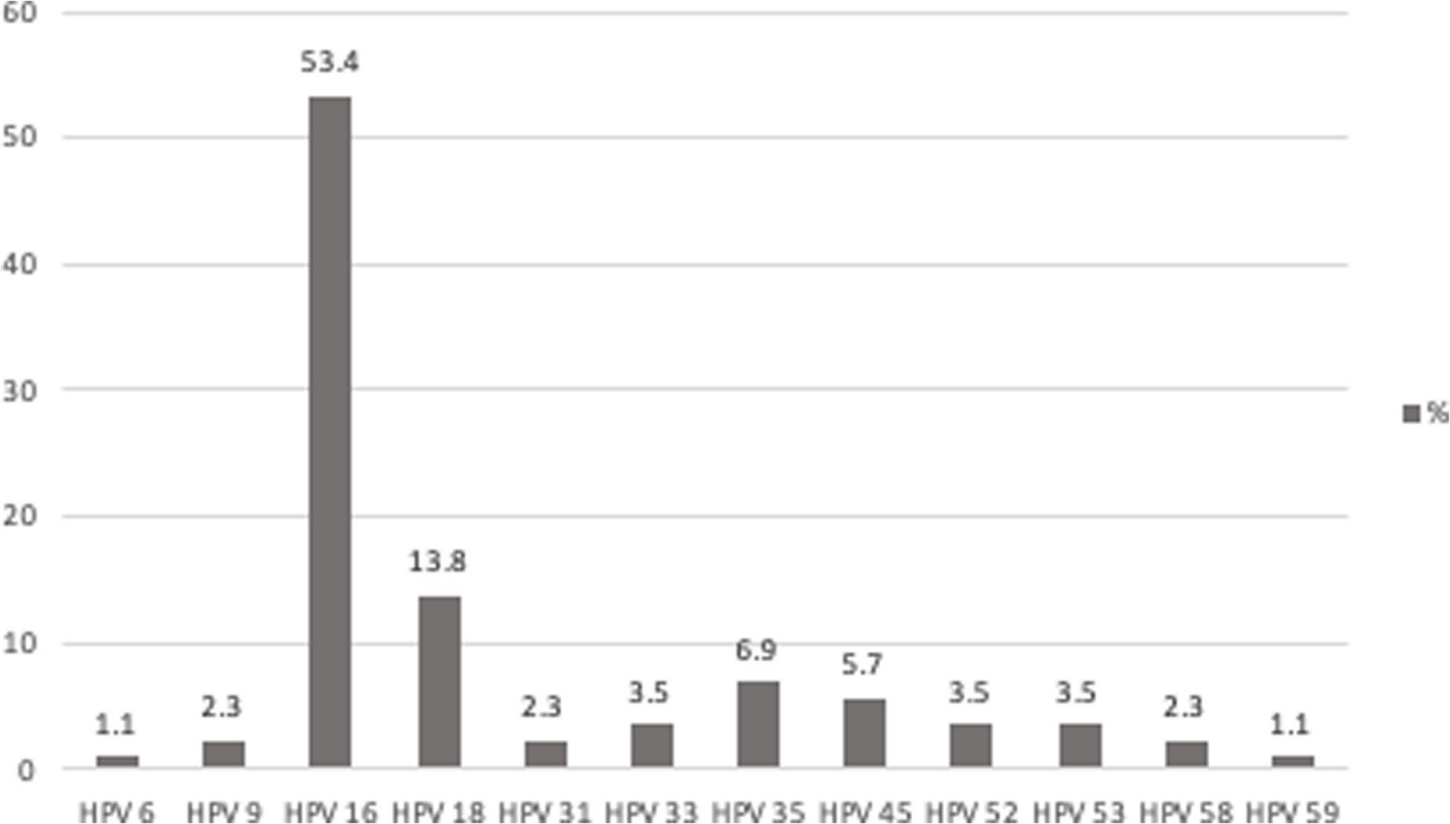
Prevalence of HPV subtypes in cervical carcinoma samples from São Luis, Maranhão, Brazil

HPV was present in 88 women (73.3%). The most prevalent type was HPV 16, which accounts for 53.4%(47/88) and combined with HPV 18 accounted for 67.2% of cases. Most of the samples had HPV types considered high risk, while less than 3.4% had HPV types considered to have low oncogenic risk (Fig. 1).

### Histological types of analyzed tumors

The most prevalent types of tumor histological types were squamous cell carcinoma with a total of 95 cases (79.1%) and 11 (9.1%) cases of adenocarcinomas. Of the 120 women in this study, 45 (37.5%) had stage IIIB tumors, in cases where HPV 16 was present, stage III was predominant with 23 samples, according to FIGO classification.

### HPV 16 variants

Of the 47 samples identified as HPV 16, 23 samples were variant A (49%), 20 (43%) were variant D, while variants B and C were present in two samples each (4%).

The mean age for women with variant A of HPV 16 was 50.6 years, 58.5 years for variant B, 58.5 years for variant C and 50 years for those with variant D (Table 5). The most prevalent histological type in tumors with HPV 16 was squamous cell carcinoma (SCC) in 38 samples, followed by adenocarcinoma in 4 samples (11%) (Table 5).

**Table 5:** Relationship between age, histological types and HPV 16 variants

Characteristics	HPV 16 variantes				
	A (*n* = 23)	B (*n* = 2)	C (*n* = 2)	D (*n* = 20)	*p* -value
**Age**					
Mean	50.6	58.5	58.5	50.0	0.624
Median	49.0	58.5	58.5	46.0	
**Histological type**					
SCC	21 (55.5)	1 (2.7)	0 (0.0)	16 (41.6)	
ADC	1 (25.0)	0 (0.0)	0 (0.0)	3 (75.0)	< 0,001
UMT	1 (33.3)	0 (0.0)	1 (33.3)	1 (33.3)	
PDC	0 (0.0)	1 (50.0)	1 (50.0)	0 (0.0)	

*SCC* squamous cell carcinoma, *ADC* adenocarcinoma, *UMT* undifferentiated malignant tumor, *CPD* poorly differentiated carcinoma

There was a statistically significant association between histological type and HPV variant (*p* < 0,001), regarding squamous cell carcinoma and HPV 16 variant “A” and “D” (Table 5).

Staging also changed depending on the HPV 16 variant. Among patients with variant A, stage III (9 patients) was predominant followed by stage II (8 cases). For those with variant D, clinical stage III (10 cases) was predominant, followed by stage II (4 cases) (Table 6).

**Table 6:** Analysis between clinical stage and HPV 16 variants

Characteristics	HPV 16 variants			
	A (*n* = 23)	B (*n* = 2)	C (*n* = 2)	D (*n* = 20)
**Clinical stage (FIGO)**				
I	4	0	0	3
II	8	0	1	4
III	9	2	1	10
IV	0	0	0	3
NI	2	0	0	0

*NI* not identified

Treatment response also depended on the HPV 16 variant. For example, 12 cases with variant A showed complete remission, compared to only 7 cases with variant D. Likewise, there were 2 deaths with variant A, and 3 who had variant D. In 12 cases it was not possible to evaluate disease status at the end of the first treatment (Table 7).

**Table 7:** Analysis between treatment response and HPV 16 variants

Characteristics	HPV 16 variants				
	A (*n* = 23)		B (*n* = 2)	C (*n* = 2)	D (*n* = 20)
**Treatment response**	Complete	12	0	1	7
	Partial	4	0	1	5
	Progression/Death	2	0	0	3
	NI	5	2	0	5

*NI* not identified

### HPV sublineages

A reference sequence described by Burk et al. [13] was used to construct the phylogenetic tree.

**Fig. 2:**
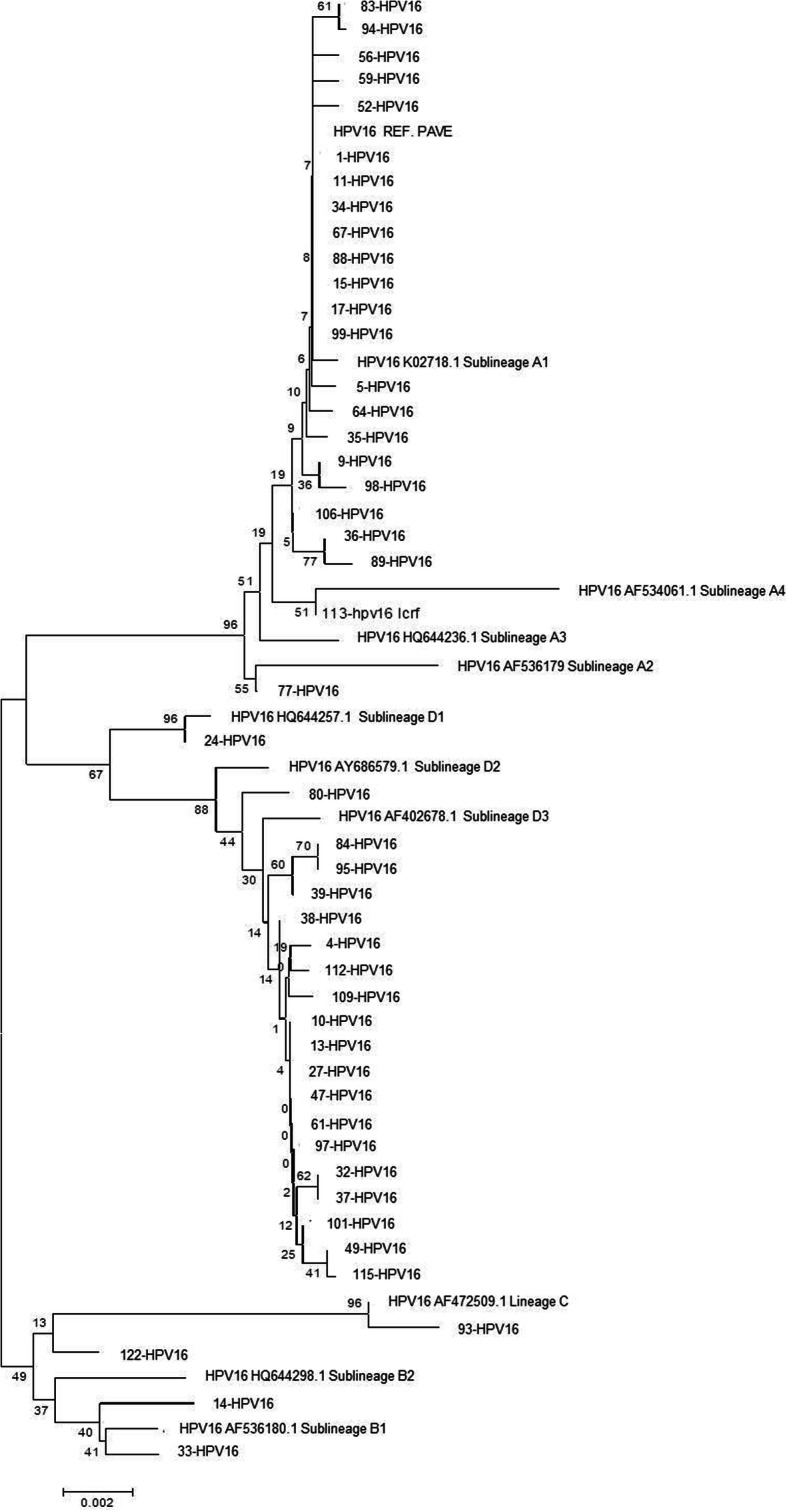
Phylogenetic tree of HPV 16 with lineages. The tree was constructed using Neighbor-Joining with *pairwise deletion*. The numbers at each node are bootstrap values (with 1000 replicates)

Among HPV 16 cervical samples classified as belonging to variant A, 21 belonged to sublineage A1, 1 belonged to sublineage A2 and 1 belonged to sublineage A4. Of the samples belonging to variant B, 1 belonged to sublineage B1 and 1 belonged to sublineage B2 (Fig. 2).

Of the HPV 16 cervical samples belonging to variant D, 1 belonged to sublineage D1, 1 belonged to sublineage D2 and 18 belonged to sublineage D3. Variant C, with 2 cases, does not have any sublineages (Fig. 2).

## Discussion

Our research evidenced that the majority of women were in the age group 40 to 49 years of age and self-declared with mixed-race, had schooling up to elementary school, had a family income between 1 and 2 times the minimum wage and were married or in a consensual union. There was no statistically significant association between the sociodemographic variables and the presence of HPV.

Cervical cancer is associated with low socioeconomic indices, presenting a higher prevalence in regions with high poverty, high illiteracy rates and precarious hygiene habits [15, 16].

A study by Wang et al. (2015) [17] reported that women who received Pap smear test tended to have higher education levels, corroborating the results of other studies, and that this may be associated with a lack of information and delays in searching for treatment. In a study conducted by Manga et al. (2015) [18] on 209 women who sought care for cervical cancer screening, the mean age was 39.6 years old, and only 15% of the women were not literate, 88% were married and 48% had a paid activity.

The virus was highly prevalent (88/120–73%) in the tumor samples examined. The prevalence of HPV in invasive cervical cancer samples can range from 70 to 100%, which may be associated with different techniques used to detect the virus [19–21].

HPV 16 is the most prevalent in cervical cancer worldwide, followed by HPV 18. However, the frequency of HPV types may vary according to the geographic region of the population under analysis. Other studies point out that types 16, 18, 31, and 45as the four most prevalent HPV types in South and Central America [13, 21].

Studies have sought to evaluate the role of intratype variants of HPV16 and HPV18 in the persistence of viral infection, the risk of cervical intraepithelial neoplasia development, and the development of invasive cervical cancer [11, 13]. For both HPV 16 and HPV 18, the distribution of variants worldwide is influenced by geographic and ethnic factors.

In the present study in São Luís do Maranhão, the most prevalent strain in the population studied was variant A for HPV 16, according to the literature. In a case-control study conducted by Hang et al. (2016) [5], we attempted to evaluate the association between HPV 16 variants and the risk of cervical cancer in 298 women with HPV 16 in China and found that variant A was predominant.

In the study conducted by Volpini et al., (2017) [22] in Brazil on 24 women positive for HPV 16, with HIV diagnosis, but anormal Pap smear, variant A accounted for 70.8% of cases (17/24), followed by the C and D variants, which together totaled 29.2% of the samples (7/24).

In a study conducted in Brazil by Vidal et al. (2016) [12] on a cohort of 594 women with invasive cervical cancer, 334 women had HPV 16. Of these, 217 (65%) belonged to variant A, 97 (29%) belonged to variant D. Variants B and C had 10 cases (3%) each.

A study by Villa et al. (2000) [23], also in Brazil, examined the geographic differences in intratype variations of HPV 16 and their associations with the development of cervical cancer precursor lesions. Variant A (54%) was the most frequent, followed by variant D in 22% of the cases. It also emphasizes the strong association between persistence and the presence of the non-European variants B, C and D when compared to the European prototype A.

The most prevalent histological type in tumors with HPV 16 was squamous cell carcinoma (SCC) in 38 samples. There was a statistically significant association between histological type and HPV variant (*p* < 0,001).

Staging also changed depending on the HPV 16 variant. Among patients with variant A, stage III (9 patients) was predominant.

Studies also indicate a variation in the distribution of HPV types among histological types. HPV 16 has been associated with squamous cell carcinoma and HPV 18 has a higher prevalence in adenocarcinoma than HPV 16. However, few studies have attempted to identify associations between HPV 16 variants and the histologic type of cervical tumors [24, 25].

In the study by Hang et al. (2016) [5], it was observed among the cases of women with cervical cancer infected with HPV 16, 289 (97%) had a pattern of squamous cell carcinoma, followed by adenocarcinoma (2.3%) and adenosquamous carcinoma (0.7%).

Treatment response also depended on the HPV 16 variant. For example, 12 cases with variant A showed complete remission, compared to only 7 cases with variant D. Likewise, there were 2 deaths with variant A, and 3 who had variant D. In 12 cases it was not possible to evaluate disease status at the end of the first treatment.

Tan et al (2019) reported that European prototype E-T350 was the most prevalent (82.76%) followed by Asian (As) variant. In patients with suspected cervical lesions the most prevalent variant was As variant (54.9%) by increasing significance with severity of cervical diseases [26].

In another study conducted by Ortiz-Ortiz et al (2015), the variants more frequently found in women with cervical carcinoma were E-G350, AA-a, AA-c, E-C188/G350 and E-A176/G350. All of them are associated with the development of cervical carcinoma, however, AA-a showed the highest association [27].

Few studies have been conducted to evaluate the effect of HPV 16 variants on the response to oncologic treatment. After analyzing 155 cases of HPV16 positive cervical cancer (132 cases of variants E × 23 cases of NE variants) Zuna et al (2011) [28] came to the conclusion that the non-European variants showed less aggressive behavior in relation to mortality than the European variants. It should be pointed out that the data should be evaluated with caution due to the small number of cases of NE variants in the study.

In our study we found a greater number of NE variants (B, C, and D). Of the 47 cases we found 23 with variant A (E) and 24 with variants B, C, and D. The mortality of patients with the NE variants was 13% (3/24), while it was 9% (2/23) in those with variant A (E). The number of cases of complete remission was also lower with the NE variants (33% × 52%). These findings can be explained by the large number of cases of variant D in our study (20 cases). A study by Burk et al (2013) [13], showed the specific aggressiveness of this variant.

We observed the predominance of sublineage A1 (21 cases), followed by sublineage D3 (18 cases). In the study by Hang et al (2016) [5], the A4 sublineage was associated with a significantly higher risk of cervical cancer than the A1-A3 sublineages (OR = 1.72, 95% CI).

In a study by Alfaro et al., (2016) [29] of 462 women with cervical cancer, HPV 16 was identified in 50.9% of the cases. Of these, the A1\2 (31.4%) sublineage was predominant, followed by the D2 (10.4%) and D3 (9.1%) sublineages. However, AA variants accounted for 38.7% of the HPV 16 positive cases.

A study by Nicolas-Parraga et al. (2017) [30], sought to explore the prevalence of HPV 16 variants in patients with cervical cancer from Europe, South-Central America, Asia and Africa. One hundred eighteen cases of squamous cell carcinoma, 120 of adenocarcinoma, and 53 of adenosquamous carcinoma were observed. Also, the highest prevalence of HPV 16 was observed in patients with squamous cell carcinoma. When examining HPV 16 variants, the A1–3 sublineages were predominant in squamous cell carcinomas (76.9 to 97% for different geographic regions) and there was a large variation for variant D depending on the geographic region in adenocarcinoma (28.6 to 63.3% for different geographic regions), and adenosquamous carcinoma (12.5 to 61.5% for different geographic regions).

The data also showed an increase in the prevalence of A1–3 in Europe (67.9 to 97% for all histological types), variant D in South-Central America (61.5 to 63%, for adenocarcinoma and adenosquamous carcinoma), A4 in Asia (from 11.5 to 27.6% for all types) and variants B and C for Africa (from 28 to 66.7% and from 12.3 to 37.5% for ECC and ADC) [30].

## Conclusions

The most prevalent HPV in cervical carcinoma samples in São Luís was HPV 16. This study evidenced for the first time the prevalence of HPV variants ant the most prevalent was variant A. There was a statistically significant association between histological type and HPV variant.

Further studies are needed to understand the role of HPV variants in the origin and progression of cervical cancer, as well as the relationship between HPV 16 and its variants with the development of precursor lesions. The data presented here may help the development of future epidemiological studies on HPV 16 variants, as well as in the creation of strategies to combat the types that remain circulating, and which were not included in the currently available vaccines against HPV.

## Data Availability

All data is included in the manuscript file. The data bank is available with the authors.

## Abbreviations

ADC: Adenocarcinoma
BLAST: Basic Local Alignment Search Tool
CACON: High Complexity Care Center in Oncology
CEP-UFMA: Research Ethics Committee of the Federal University of Maranhão
CESC-UEMA: Center of Advanced Studies of Caxias of State University of Maranhão
DNA: Deoxyribonucleic acid
FAPEMA: Support Foundation for research and scientific and technological development of the State of Maranhão
FIGO: International Federation of Gynecology and Obstetrics
HPV: Human papillomavirus
ICF: Informed Consent Form
INCA: National Institute of Cancer
HUUFMA: University Hospital From Federal University of Maranhão
LCR: Long control region
MEGA: Molecular Evolutionary Genetics Analysis
PCR: Polymerase chain reaction
PDC: Poorly differentiated carcinoma
RNA: Ribonucleic acid
SCC: Squamous cell carcinoma
UMT: Undifferentiated malignant tumor
